# Could Moral Enhancement Interventions be Medically Indicated?

**DOI:** 10.1007/s10728-016-0320-8

**Published:** 2016-02-24

**Authors:** Sarah Carter

**Affiliations:** The Centre for Social Ethics and Policy, School of Law, University of Manchester, Williamson Building, Oxford Road, Manchester, M13 9PL UK

**Keywords:** Moral enhancement, Bioethics, Neuroethics, Moral therapy, Empathy, Enhancement

## Abstract

This paper explores the position that moral enhancement interventions could be medically indicated (and so considered therapeutic) in cases where they provide a remedy for a lack of empathy, when such a deficit is considered pathological. In order to argue this claim, the question as to whether a deficit of empathy could be considered to be pathological is examined, taking into account the difficulty of defining illness and disorder generally, and especially in the case of mental health. Following this, Psychopathy and a fictionalised mental disorder (Moral Deficiency Disorder) are explored with a view to consider moral enhancement techniques as possible treatments for both conditions. At this juncture, having asserted and defended the position that moral enhancement interventions could, under certain circumstances, be considered medically indicated, this paper then goes on to briefly explore some of the consequences of this assertion. First, it is acknowledged that this broadening of diagnostic criteria in light of new interventions could fall foul of claims of medicalisation. It is then briefly noted that considering moral enhancement technologies to be akin to therapies in certain circumstances could lead to ethical and legal consequences and questions, such as those regarding regulation, access, and even consent.

## Introduction

In her 2013 paper, Casal [9, p. 2] suggests that we could define the goals of moral enhancement interventions in terms of a satiable requirement, such as reducing crime or even “elimination of wrongdoing”, and then makes the case that if we are to take this view then, in this context, this would be akin to a case of moral *therapy*. She explains that this is because the use of the endeavour towards such a goal “aims at eliminating pathologies or shortfalls from an appropriate threshold of compliance. This option will involve correcting those individuals with a deficit of empathy or an excess of aggression…” [9, p. 2].

Casal makes an interesting point, although one which in turn raises the question as to whether moral enhancement interventions *could* be regarded as therapeutic, rather than merely enhancing, in certain circumstances. In this paper, I will consider the idea that moral enhancement interventions could be medically indicated (as a treatment) in a more general sense than that suggested by Casal, and explore whether it could perhaps work in a more medical setting.^1^

The question as to whether moral enhancement techniques could be medically indicated (and so therapeutic) is one that is important to consider as it could have far-reaching consequences. Regarding an intervention as a treatment or therapy in certain circumstances will raise new questions for that treatment regarding, for instance, its regulation, people’s access to it, as well as questions regarding consent, and when it is appropriate to offer the treatment.^2^ I will address these in a little more detail later in the paper.^3^

The main objective of this paper is to attempt to answer the question of whether ‘moral enhancement interventions could be medically indicated’, and in consideration of this question, I briefly explore some of those consequences noted above. In order to answer the central question, I will first outline what is meant by the term ‘moral enhancement’ for our purposes, and also by the term ‘empathy’ as this is what would most likely be addressed in moral enhancement interventions [30]. From there, I will raise the question as to whether a deficit of empathy could be considered pathological, offering two possible cases for reference: Psychopathy, and the fictionalised disorder of Moral Deficiency Disorder. Thereafter I will note questions regarding medicalisation that could be raised as this juncture, before returning to the central question (whether moral enhancement interventions could be medically indicated) and commenting on the possible consequences and questions that could arise.

## Defining Moral Enhancement and Empathy

It would perhaps be prudent to clarify now what is meant by the term ‘moral enhancement’ for our purposes, as there are of course numerous accounts which offer suggestions as to what the endeavour could involve. Some of the most prominent voices in this discussion are Harris [17], who favours a cognition-centred approach to moral enhancement, Persson and Savulescu [30] who argue that the endeavour involves an increase in levels of empathy, and Douglas [14], who argues that moral enhancement interventions are those which would attenuate counter-moral emotions. In this paper, I will use the account of moral enhancement offered by Persson and Savulescu: that moral enhancement inventions are those which increase levels of empathy.

If moral enhancement involves increasing levels of empathy, as Persson and Savulescu suggest, then it would be reasonable to assume that a deficit of empathy would be the thing that moral therapy interventions would address. I will return to this assertion in the sections that follow, but first, given the discussion at hand it would be prudent to question what is meant when we speak of empathy. Perhaps surprisingly, this question is not as easily answered as one might expect, for as Hodges and Klein note: “There are almost as many definitions of empathy as there are researchers who have studied the topic” [18, p. 438]. Due to space constraints, I will focus my attention principally on the definition of empathy presented by Persson and Savulescu and Simon Baron-Cohen.

Persson and Savulescu define empathy as “a capacity to imagine vividly what it would be like to be another, to think, perceive, and feel as they do” [30, p. 111], as such—they argue—it has no motivational component. Rather, empathy in this sense is simply a component of altruism, which in turn has the motivational component of sympathetic concern for the feelings and well-being of others. But Persson and Savulescu note later that the term could also be used in an extended sense, “such that empathy includes sympathy or a concern for the well-being of others, not merely imagining what the experiential state of another is like” [30, p. 116].

They note further that it is in this extended sense that the term is used by Baron Cohen, whose definition of empathy progresses in a similar way to that of Persson and Savulescu. Baron-Cohen begins his definition of empathy by stating that:

Empathy occurs when we suspend our single-minded focus of attention, and instead adopt a double-minded focus of attention.

‘Single-minded’ attention means we are thinking only about our *own* mind, our current thoughts or perceptions. ‘Double-minded’ attention means we are keeping in mind *someone else’s* mind, *at the very same time*…. When empathy is switched off, we think only about our own interests. When empathy is switched on, we focus on other people’s interests too [3, p. 10].

Baron-Cohen then goes on to extend his definition of empathy:

Empathy is our ability to identify what someone else is thinking or feeling, and to respond to their thoughts and feelings with an appropriate emotion.

This suggests there are at least two stages in empathy: recognition and response. Both are needed, since if you have the former without the latter you haven’t empathised at all… Empathy therefore requires not only that you can *identify* another person’s feelings and thoughts, but that you *respond* to these with an appropriate emotion too [3, p. 11].

It is in this second part of Baron-Cohen’s definition that the motivational component of empathy becomes apparent—and it is this extended definition,^4^ nodded to by Persson and Savulescu and explicitly defined by Baron-Cohen, that will serve as the working definition of empathy for this paper.

Persson and Savulescu [30] argue further that moral enhancement is required in order to prevent mankind from bringing about an instance of ultimate harm; as they put it: “a heightened moral sensitivity is necessary to reverse this descent of humanity down a spiral of ever-increasing existential risks” [29, p. 666]. For this reason then, the endeavour could be said to be beneficial for all concerned. Further, even if considered in less dramatic terms, the benefits of living in a society which has been morally enhanced could quite easily be imagined, for as Jebari [20] notes: “empathetic people avoid harming others, are more willing to cooperate with strangers, and are more willing to benefit others”.

It is important to note that unlike in the cases other enhancements of, for example, cognition, memory, strength, etc., moral enhancement interventions do not immediately appear to confer benefits to the enhanced individual *directly*. If one is a part of a morally enhanced society then the benefits of the endeavour are clear, but on an individual level they may not be so apparent. This is particularly problematic for our purposes as those for whom moral enhancement interventions may be considered medically indicated might be disinclined to undergo the treatment as they could be unlikely to see a benefit for themselves in doing so, especially if they already consider their deficit of empathy to be an advantage in their day-to-day lives. This assertion could in part be argued from common sense: if a person’s life is made easier by their reduced experience of empathy, then they might not see an enhancement of that trait to be a sensible course of action. This point is alluded to by Kevin Dutton, who found that many surgeons scored highly on tests used to identify psychopaths; he noted:

The most important thing when you’re conducting a dangerous operation, a risky operation, is you’ve got to be very cool under pressure, you’ve got to be focused. You can’t have too much empathy for the person that you’re operating on, because you wouldn’t be able to conduct that operation [35, p. 374].

It would not then be a great leap to imagine that those whose lack of empathy benefits their (for example) career in business, medicine, finance, or indeed crime might be less-than-enthusiastic about the idea of having their levels of empathy increased and risking losing that edge. Further, writers such as Baron-Cohen [3] and Hodges and Klein [18] have noted that maintaining even slightly above-average levels of empathy can prove emotionally costly and exhausting, and so perhaps this fact might make moral therapy again appear a less-than-desirable option to those who we might consider to need it.

So then, if a deficit of empathy can be seen as pathological (and so moral enhancement interventions could be medically indicated in such instances),^5^ much would centre on whether people with that disorder considered themselves as being harmed by having it—and so therefore whether treatment would be seen as a benefit or a burden.

Casal believes that people would consider moral therapy to be a benefit; as she puts it:

…if we see moral compliance as a benefit, and lacking empathy or becoming a criminal as personal misfortunes, those who need biotherapy to become as good as others also have a complaint if biotherapy is denied to them, thereby depriving them of what others have. There are thus also egalitarian arguments in support of moral biotherapy… [9, p. 3].

This being said, it is not entirely clear how people would feel about their lack of empathy and, in turn, moral therapy. It could be that Casal is correct and that therapy would be seen as beneficial by the person that we would consider in need of it; or indeed it could be the case that those with lower levels of empathy would see any attempt to correct that as a burden, something which they would much rather avoid.^6^

However, there could perhaps be claims made that treatment could still benefit such people directly, in addition to the indirect advantages already mentioned. This is because, if an increased level of empathy were to lead to a reduction in wrongdoing (as would of course be a desired (if not expected) outcome), then this in turn would mean that the person in whom the endeavour was medically indicated would be less likely to engage in activities that could result in consequences that would be undesirable for her in particular. This could involve being subject to legal retributivism tactics such as fines, community service, or jail time, or indeed (perhaps more sinisterly) becoming the victim of consequences outside of the law (for instance, gang related-violence, bar fights, assault, etc.).^7^ And so while the advantages of accepting moral enhancement interventions as treatment might not be immediately clear to the individual in whom it may be medically indicated, it is not the case that the endeavour would be without benefit for her.

## Defining Treatment, Disease and Disorder

Before asking whether moral enhancement interventions could be considered treatments (in those circumstances where they would be medically indicated), it would perhaps be prudent to first clarify what is meant by the term ‘treatment’. Daniels defines treatment as “services or interventions meant to prevent or cure (or otherwise ameliorate) conditions that we view as diseases” [13, p. 309].^8^ So then in order to answer the question as to whether moral enhancement techniques could, in certain contexts, be considered therapeutic, one would have to demonstrate that there existed something—some disease or disorder—for which moral enhancement interventions would be considered a treatment; a matter I will return to later. However, as Resnik [31] notes, there is no single, agreed-upon definition of health from which we can derive our understanding of ‘disease’. He explains that there are two basic approaches to the definition of health: the value-neutral (descriptive) approach, and the value-laden (normative) approach.

The value neutral approach considers health to be an empirical, descriptive concept which is based on factual information about human biology and normal human functioning. Arguably the best-known, most influential account of this approach to health comes from Boorse [7], who asserted that in this context the term ‘normal’ referred to species-typical: those traits that are statistically typical for members of that species to have.^9^ So then as Resnik explains: “a human with healthy lungs has specific respiratory capacities that are normal in our species… A human who lacks these capacities, such as someone with cystic fibrosis or emphysema, has a disease” [31, p. 366].

Meanwhile, the value-laden approach bases concepts of health and disease on societal, cultural, and even moral norms; so then a person that falls within such norms is considered healthy, whereas another person who does not is considered diseased. So then, as Resnik [31, p. 367] notes, a person who “deviates from species-typical functions could be considered healthy in a society that views that deviation as healthy”.

So it is not necessarily clear as to what constitutes health—nor, in turn, a disease—even in the realms of physical health; and it is unlikely to be much clearer when considering cases of mental health. This is thrown into sharp focus when we consider that there is little clarity or even agreement [28] with regards to what constitutes a mental (as opposed to physical) disorder.

In English law the answer to this question remains unclear: the only definition of mental disorder offered in the Mental Health Act is extremely wide, defining it simply as meaning “any disorder or disability of the mind” [26].

Even the Diagnostic and Statistical Manual of Mental Disorders (DSM), which provides standard criteria for the classification of mental disorders, precedes its own description by admitting that no definition of ‘mental disorder’ adequately encapsulates the complexity of the concept. This is particularly clearly noted in DSM-IV:

Although this manual provides a classification of mental disorders, it must be admitted that no definition adequately specifies precise boundaries for the concept of mental disorder… Mental disorders have… been defined by a variety of concepts (e.g. distress, dyscontrol, disadvantage, disability, inflexibility, irrationality, syndromal pattern, etiology, and statistical deviation). Each is a useful indicator for a mental disorder, but none is equivalent to the concept, and different situations call for different definitions [as cited in 6, p. 165].

This is echoed in the most recent edition of the text, DSM-5, which states that “no definition can capture all aspects of all disorders in the range contained in DSM-5” [2, p. 20]. Despite this, both DSM-IV and DSM-5 do still offer an attempt at a definition of mental disorder, with the aforementioned disclaimers in mind:

A mental disorder is a syndrome characterised by clinically significant disturbance in an individual’s cognition, emotion regulation, or behaviour that reflects a dysfunction in the psychological, biological, or developmental processes underlying mental functioning. Mental disorders are usually associated with significant distress or disability in social, occupational, or other important activities. An expectable or culturally approved response to a common stressor or loss, such as the death of a loved one, is not a mental disorder. Socially deviant behaviour (e.g. political, religious, or sexual) and conflicts that are primarily between the individual and society are not mental disorders unless the deviance or conflict results from a dysfunction in the individual as described above [2, p. 20].

## Could a Deficiency of Empathy be Pathological?

So with the DSM-5 definition in mind, *could* a lack of empathy be considered a case of mental disorder? Perhaps so—given the neurological basis of empathy [27] it could be argued that a deficit of empathy could be demonstrative of “a dysfunction in the psychological, biological, or developmental processes underlying mental functioning” [2, p. 20]. Further, this deficit of empathy could affect moral decision-making^10^ and in turn behaviour—fulfilling two of the key criteria listed above for defining a mental disorder.

As a deficit of empathy could be considered a mental disorder under this definition—and as a deficit of empathy would most likely be treatable by increased levels of empathy—then we could consider any condition characterised by such a deficiency to be a candidate for treatment through moral enhancement techniques. So then, in such instances, moral enhancement interventions could indeed be medically indicated.

When considering whether such a condition could exist, one well-established disorder comes to mind: psychopathy; however the position that empathy lies at the core of this disorder is one that is somewhat disputed [23]. Nevertheless a condition characterised by a lack of empathy—that moral enhancement techniques could treat—could indeed exist, but is not currently classified among existing mental disorders.^11^

Later in this paper I will consider a hypothetical new condition: Moral Deficiency Disorder. This disorder would be characterised by a deficit of empathy and would principally be diagnosed in those individuals whose capacity for moral reasoning and action would benefit significantly from an increased level of empathy—that is, those for whom moral enhancement interventions would be medically indicated and considered a treatment.

In the sections that follow, I will consider these possible candidates for treatment by moral enhancement techniques—psychopathy and Moral Deficiency Disorder—in turn.

## Psychopathy

Lockwood et al. [22, p. 1] note that: “Psychopathy is a disorder characterized by a lack of empathy, shallow affect, and manipulation of others for own gain”.

On this view of psychopathy as a condition with a lack of empathy at its very core, it would make sense to put forward moral enhancement interventions as a treatment (thus making it an instance of moral therapy). For if an increase in the levels of empathy in a psychopathic individual would cure that psychopathy, or at the very least temper the symptoms thereof, it then seems reasonable to consider the endeavour to have a therapeutic effect in this instance. However, the position that empathy is at the core of psychopathy is not without its critics.

One such critic is Maibom, who argues that the way that empathy is measured is flawed, with heavy reliance on self-reporting which is open to issues such as social desirability and stereotyping. For example, Maibom [23] notes that studies have shown that women who are aware of being observed tend to score as having higher levels of empathy than in studies where they are unaware of being observed (in these cases, women and men demonstrate equal levels of empathy). Glenn et al. [15] note as well that the issues surrounding the use of self-report methods are compounded by the fact that psychopaths tend towards dishonesty. Given our “blunt tools” for measuring empathy, Maibom [23, pp. 98–99] asserts: “This means that there is little support for theories linking psychopathic immorality directly to emotions that are usually regarded to be *moral* emotions, such as empathy and sympathy”.

A study by Blair [4], however, suggests that there is something missing in psychopathic moral decision-making and that thing does seem to be, if not empathy, then certainly something closely related. Blair tested preschool children to see whether they could distinguish between social conventions (e.g. wearing outdoor clothes indoors) and moral rules (e.g. hitting another pupil); he found that the children saw moral transgressions as more serious than social ones. Further, when asked to explain why an action was wrong, children said “those are the rules” in regards to social conventions, but when speaking of moral rules the children made reference to the wellbeing of others. Finally, when asked whether they would consider an action to be acceptable if their teacher (or other authority figure) had permitted it, the children agreed that the acceptability of social conventions could be altered in this way, but disagreed that this could be the case in the instance of moral rules.

Blair then repeated the study but this time with incarcerated psychopaths (using an equivalent number of incarcerated nonpsychopaths as a control). Blair found that, unlike the children, the psychopaths did not consider moral transgressions to be more serious than those against social conventions (or vice versa). Also, when asked to explain why an action was wrong the psychopaths made reference to “the rules” for both moral and social transgressions and did not seem to consider the welfare of others in their reasoning.^12^ These findings have since been replicated with a larger number of respondents [5].

That the psychopaths did not make reference to the welfare of others in their reasoning does then seem to suggest that empathy may indeed be at the heart of their condition. As Adshead puts it: “Psychopaths demonstrate failure/lapses in moral reasoning when they harm others; psychopaths have emotional deficits; ergo emotional deficits are relevant to failures in moral reasoning” [1, p. 119]. But is that deficient emotion empathy? It is noted [1, 23] that psychopaths also have a dramatically reduced experience of fear compared to non-psychopaths; this is certainly something which could go some way to explain their reduced sensitivity to the threat of punishment for wrongdoing [15] (especially if the act in question involves a reward), but it seems less clear that this could be the missing factor in their inability to reason morally. As Adshead notes: “Empathy is relevant to moral reasoning and makes explicit the role of personal emotional experience in moral decision making” [1, p. 120].

I do not have the space to question and consider the role played by this deficit of fear in the psychopaths’ inability to reason morally in the depth that it deserves, but this does at least demonstrate that the view that empathy is at the heart of this condition is not without its critics and questions. Consequently the idea that moral enhancement techniques could be used to treat psychopaths might well be supported by many, but it would not be uncontroversial, and given the controversy surrounding the role of empathy in psychopathy, it may be worth putting the issue to one side for now. It is therefore necessary to explore whether there could be an alternative approach to the matter at hand.

## Moral Deficiency Disorder

Imagine that we were to identify a group of individuals whose capacity for moral reasoning and action would benefit significantly from an increased level of empathy; such people could be said to suffer from—to give it a name—Moral Deficiency Disorder (or MDD), for which, moral enhancement techniques could clearly be considered appropriate treatment. Yet this hypothetical proposal is not without its problems.

First, it is not clear how we would identify those individuals who would be considered as suffering from MDD. At present, the typical method used to identify levels of empathy is by using fMRI to identify activity in specific areas of the brain. As Adshead notes: “Measures of empathy have been developed, and the neural basis of empathy is thought to involve a complex set of neural networks involving the limbic system, hippocampus, and orbital frontal cortex” [1, p. 120]. But scanning the brains of an entire populace seems an excessive (as well as costly) undertaking, so the question could be raised: when would it be appropriate to test for MDD?

As noted earlier in the paper, a diagnosis for a mental disorder characterised by a lack of empathy would likely be reliant on those with such a deficiency displaying certain behaviours as a result of that deficit. So then it may be the case that one might consider focusing our attention on providing moral enhancement techniques as treatment for those who show a deficiency of empathy. And as it seems a sensible course of action to deal with the condition early so as to hopefully reduce (or indeed remove) the risk of harm to others in the future, a case could therefore be made for turning our attention to children whose behaviour is symptomatic of a deficit of empathy. However, targeting children in this manner is problematic, as it isn’t clear whether a certain child will retain this deficiency into adulthood, or whether she will simply grow out of it. As Seifert [34] notes: “Some of the traits seen in a psychopath—such as lack of empathy, little or no social respect, and disregard for moral boundaries—are the same traits seen during infancy and very early childhood”.^13^ So diagnosing MDD in children would be an inexact science and would likely cause serious issues as regards informed consent, given that we would likely be unable to tell whether the child’s empathy deficiency is a marker for a long-term condition, or simply a sign of the child developing at a slightly different rate. Further, the concern could be raised that the label provided by an MDD diagnosis could be considered rather stigmatising; this is particularly problematic in the context of childhood diagnoses of MDD, as this would be conferring a stigmatising label at an early age.^14^

This is not necessarily to say that children exhibiting behaviour symptomatic of MDD should be ignored unless that behaviour continues into adulthood, but it is important to note those practical and ethical issues involved in treating minors in such a situation. Perhaps the best that we could do in such cases would be to monitor the children in question in the hope of being able to offer treatment at a later stage should the symptoms of MDD persist into their adult years.^15^

Unfortunately, treating adults with an empathy deficiency would be fraught with practical and ethical problems as well. First of all, as noted at the start of this section, it would be impractical to attempt to scan the brains of all adults in a given populace in the hope of locating those with a deficit of empathy, and so we would have to target particular persons in order to use this method efficiently. Following the above discussion, the clearest group of candidates would of course be those people who exhibited behaviour indicative of a deficit of empathy throughout their childhoods and who continue to do so into adulthood. Another group of candidates could perhaps be offenders; there may of course be some overlap between this group and those whose behaviour in childhood (continuing into adulthood) indicates a deficit of empathy.

Such targeting raises issues of its own—in the case of offenders, for instance, there may be concerns regarding coercion, even if their consent seems to be freely given.^16^ Furthermore, it is not entirely clear that people with MDD would be inclined to undergo moral enhancement interventions in any instance, despite the benefits that it might provide.^17^ This is not to say that offenders, and indeed adults generally, identified as having MDD would invariably refuse to undergo moral enhancement interventions as treatment, but it is important to acknowledge that the voluntary uptake of that treatment might not be very high.^18^

While it is therefore unclear that a targeted approach would prove effective in either diagnosing MDD or providing moral enhancement interventions as therapies to those that have it, this does not diminish the fact that moral enhancement technologies could, in principle, constitute a treatment for such a condition.

This widening and shifting of medical definitions to allow for a wider range of diagnoses—based on new information or, as in this case, new possible treatments –could perhaps be an instance of medicalisation. As Peter Conrad writes:

Virtually any human difference is susceptible to being considered a form of pathology, a diagnosable disorder, and subject to medical intervention. As Nancy Press notes, “Medicalisation pathologises what might otherwise be considered as simply variations in normal human functioning” [11, p. 148].

In the section that follows, I will explain what is meant by medicalisation, and also briefly consider whether the introduction of something such as Moral Deficiency Disorder would constitute an instance of medicalisation for social control.

## Medicalisation

Conrad [10, p. 211] defines medicalisation as consisting of “defining a problem in medical terms, using medical language to describe a problem, adopting a medical framework to understand a problem, or using a medical intervention to “treat” it”. Medicalisation can also involve the widening of diagnostic criteria for already-established illnesses or disorders, in turn increasing the number of diagnoses for that condition [11].

Schermer notes that an important factor in the process of medicalisation is the availability of treatment (or at least relevant medication). Discussing her research into ADHD, she writes:

Interestingly, one of the most important reasons our respondents gave for considering ADHD a disorder was the fact that there was medication for it: “Well, because there is medication for it so, yes, then I think you really have something. Because you would not take medication for nothing,” said one respondent. Having a disorder legitimised the use of medication (‘you would not use it for nothing’) but at the same time, medication itself functions as a proof for the existence of a disorder [33, p. 33].

She notes further that this circular reasoning is also found in experts: “a positive reaction to a trial of psycho-stimulant medication is often considered to be confirmation of the diagnosis… If a trait or function can be improved, it must have been defective before, this type of reasoning suggests” [33, p. 33–34].

This way of thinking could clearly be attributed to the use of moral enhancement interventions as treatments for Moral Deficiency Disorder. That is, there is a medication (moral enhancement interventions) that can improve a function (empathy-based moral reasoning/action) therefore “it must have been defective before”.

One could therefore be concerned that medicalisation is linked to the pharmaceutical industry. For instance, reporting a conversation with Bob Hare—inventor of the Psychopathy Checklist diagnostic tool (wherein a score of 30+ prompts a diagnosis of psychopathy)—journalist Jon Ronson notes the psychiatrist’s concern that the diagnostic goalposts would change if a treatment for psychopathy were discovered:

‘Sure,’ Bob said, ‘over-labelling occurs. But it’s being perpetrated by the drug companies. Just wait and see what happens when they develop a drug for psychopathy. The threshold’s going to go down, to twenty-five, twenty…’ [32, p. 283].

The view that medicalisation is linked to the pharmaceutical industry is not, however, one that is shared universally; Conrad seems particularly sceptical of this view. He notes that:

…in virtually all studies where they were considered, the corporate players in medicalisation were deemed secondary to professionals, patient movements, or other claim-makers. By and large, the pharmaceutical and insurance industries were not central to the analyses [11, p. 10].

Rather, he asserts that there are many areas from which medicalisation can emerge—but focuses on an area which seems particularly relevant for our purposes: medicalisation of deviance to provide medical social control.

A rather extreme example of medicalisation for the purposes of social control that comes to mind is Drapetomania [36]—the mental illness that a slave was said to have if she had a tendency to try to escape from her master. This is a clear example of the use of medical definitions as a tool for maintaining society in a certain way. Given that no such extreme^19^ examples remain, one could be forgiven for thinking that medicalisation for social control was no longer an issue. However, Bolton notes that we can still see the use of medicalisation—particularly in the domain of psychology—as a means of social control, especially in those cases where the patient is reluctantly brought to the attention of medical professionals as opposed to having presented himself freely. As he puts it:

Focus on the individual best fits the traditional doctor-patient model in physical medicine: a patient in distress presents themself and seeks treatment. The other kind of case, in which an individual is brought by others, the reluctant patient with no complaints of their own, is a more problematic fit with the medical model, apparently suiting more the construal of psychiatry as a form of social control [6, p. 229].

There is an attempt to defend against the use of classifications of disorder (specifically those of a psychological nature) for the purposes of social control in DSM-5:

“Socially deviant behaviour (e.g. political, religious, or sexual) and conflicts that are primarily between the individual and society are not mental disorders unless the deviance or conflict results from a dysfunction in the individual as described above” [2, p. 20].

Whilst this does seem to protect against wildly specific mental illnesses such as Drapetomania being invented solely as a means of social control, it could, however, perhaps still leave us open to more complicated questions. To illustrate this, I will briefly consider whether Moral Deficiency Disorder could be considered an example of medicalisation for the purposes of social control.

Moral Deficiency Disorder (or MDD) is characterised by a deficit of a specific emotion—empathy—that can impact on behaviour and also moral decision-making (an area of cognition). As such, it could be considered a mental disorder under the DSM-5 definition, regardless of its impact on society. But, as already noted, for those who have the disorder, MDD might prove not to be a burden or problem in their lives as a whole; indeed it might actually help those with the disorder to live the life that they want, unburdened by concern for others. So even if we do consider MDD to be a legitimate mental disorder, there is a chance that treatment would more likely be pursued for the benefit of society than for the benefit of the person with the disorder. If such an assertion is correct, this would of course raise important questions regarding the acceptability of such a practice; not least due to the implications that this could have for autonomy and consent. However, as already noted in an earlier section, there *are* benefits, both direct and indirect, to be gained from the intervention, so the concern may in fact be unfounded. Unfortunately I do not have the space here to consider this in the depth that it deserves, but it is worth acknowledging that using moral enhancement interventions as therapy could open the door to considerable criticism.

## Consequences of Moral Enhancement Interventions as Therapy

To return to the focus of this paper, it therefore seems that moral enhancement interventions could be medically indicated. I have demonstrated that a deficit of empathy could be considered pathological, and, further, as moral enhancement interventions would increase levels of empathy, it is reasonable to suggest that these interventions would, in such instances, constitute a therapy or treatment.

Therefore the question could be raised as to what consequences would arise if moral enhancement interventions were medically indicated, and so considered therapeutic. As answering this new question is not the purpose of this paper, I will consider it only briefly, but it is still a very relevant question given the assertion above.

As noted at the start of this paper, considering moral enhancement interventions as treatments could raise questions regarding concerns such as regulation, access, consent, and even when to intervene. Regulation would become an issue here, as treatments would most likely be regulated differently to enhancements when the latter comes into public use more readily [19]. A related issue is that of access to moral enhancement or indeed therapy interventions. If moral enhancement interventions were to be considered therapeutic under certain circumstances, access to those interventions (as therapies) could be dramatically altered; for instance, it might be readily available as a therapy but not as an enhancement. An example of such a policy in action can be seen in case of Ritalin (methylphenidate). Ritalin is prescribed as a treatment for ADHD but is considered an illegal substance (a class B drug) [16] for those without the condition who wish to take it as an enhancement to aid concentration.^20^

The psychological nature of the disorders which moral enhancement interventions would most likely treat means that questions regarding consent could be raised in considering moral enhancement interventions as therapeutic, as mental health treatment is, of course, the only context within which otherwise competent adults can be treated without their consent under certain circumstances [25].

And finally, as noted above, there will of course be the need to raise questions with regards to *when* it is appropriate to intervene with moral therapy: during childhood, when questions regarding development and informed consent come to the fore, or perhaps for adults with low empathy? Or is intervention more appropriate in the case of offenders? But here as well there are problems regarding free consent and indeed the fact that people may simply be uninterested in undergoing moral enhancement techniques, even if, in their case, they would be therapeutic.

## Conclusion

I have explored how a lack of empathy could be considered pathological and so something that would treat this deficiency—in this case, moral enhancement techniques—could indeed be considered medically indicated, and so a treatment. This assertion, however, raises a number of ethical concerns—as we have seen. Questions regarding the medicalisation of morality (by way of medical social control) come to the fore, as do other concerns regarding regulation and consent among others.

Considering moral enhancement interventions as medically indicated (and therefore therapeutic) in some instances therefore opens up the field to many other questions and areas of debate. It was not my intention to consider these questions and issues in this paper, but given that I have shown that moral enhancement interventions could be medically indicated,^21^ there is now space in the literature for these areas to be explored in this new context.

## Footnotes

1 To clarify: I will be using the terms ‘enhancement’ and ‘therapy’/’treatment’ as shorthand for bioenhancement, biotherapy, and bio-treatment.

2 I will use the terms treatment and therapy interchangeably in this context.

3 It is not the purpose of this paper to dwell on these issues; however it is important to acknowledge the consequences of considering moral enhancement interventions as therapeutic (within certain contexts).

4 Understanding empathy in this extended sense is not without its critics, in particular Coplan [12]; however this extended account of empathy is widely used in the literature in any case (as Coplan acknowledges).

5 As I shall argue in the sections to follow.

6 Further, there could also be concerns regarding a loss of identity; I do not have the space to discuss this issue here, however I have explored this in some detail previously [8].

7 Of course this is not to say that people without a deficit of empathy never find themselves in such situations, however it is reasonable to assume that aggressive people, and those who participate in criminal acts, are more likely to encounter such consequences with much more frequency than less aggressive people who do not.

8 This in turn could raise the question of what we could consider to be a disease; I shall consider this in a short while.

9 It is prudent to note that one could argue that even Boorse’s account involves some normative judgement, as departure from species typical functioning is considered undesirable; however, this is beside the point for our purposes. (I am grateful to the anonymous reviewer for bringing this to my attention).

10 This assertion is explored in more detail in the section on psychopathy.

11 This is noted as well by Simon Baron-Cohen [3, pp. 107–110].

12 Although, interestingly, the psychopaths did seem to consider both social conventions and moral rules to be authority-*in*dependent; that is that even if a figure of authority were to permit that action, it would still in actuality be wrong to perform it—although Blair seemed to dismiss this as the psychopathic inmates hoping to show that they had reformed and “learned the rules” rather than an actual assertion of belief.

13 I will concede that this assertion does seem to contradict the findings of Blair noted above. Unfortunately I do not have the space to explore this issue further, but I would still be inclined to argue that diagnosing children with MDD would most likely be an inexact science due to the nature of childhood development more generally.

14 I am grateful to the anonymous reviewer for bringing this point to my attention.

15 If we were to find a genetic marker which indicated a lack or deficit of empathy, then this could go a long way to assisting us in diagnosing MDD in both children and adults; however it is beyond the scope of this paper to explore this idea (and its related ethical concerns) in sufficient depth.

16 This issue is explored in some detail in [24].

17 As noted in the section on defining moral enhancement and empathy.

18 I explore this briefly in [8].

19 Although it is doubtful this was considered as such when it was in common usage.

20 This issue was explored in a report by BBC News in 2008 [21].

21 As was my intention in this paper.
